# Angiogenic Potential of Co-Cultured Human Umbilical Vein Endothelial Cells and Adipose Stromal Cells in Customizable 3D Engineered Collagen Sheets

**DOI:** 10.3390/jfb13030107

**Published:** 2022-07-29

**Authors:** Philipp Nessbach, Sascha Schwarz, Tanja D. Becke, Hauke Clausen-Schaumann, Hans-Guenther Machens, Stefanie Sudhop

**Affiliations:** 1 Department for Plastic Surgery and Hand Surgery, Klinikum Rechts der Isar, Technical University of Munich, 81675 Munich, Germany; philipp.nessbach@tum.de (P.N.); hans-guenther.machens@mri.tum.de (H.-G.M.); 2 Center for Applied Tissue Engineering and Regenerative Medicine (CANTER), University of Applied Sciences Munich, 80335 Munich, Germany; sascha.schwarz@gmx.de (S.S.); tanjabecke@googlemail.com (T.D.B.); hauke.clausen-schaumann@hm.edu (H.C.-S.); 3 Center for Nanoscience (CeNS), Ludwig Maximilian University of Munich, 80539 Munich, Germany

**Keywords:** regenerative medicine, tissue engineering, tissues and organs, biomaterials, 3D biomimetic scaffolds, cell–biomaterial interface, wound healing, 3D collagen sheet, human umbilical vein endothelial cells (HUVECs), adipose stromal cells (ASCs)

## Abstract

The wound healing process is much more complex than just the four phases of hemostasis, inflammation, proliferation, and maturation. Three-dimensional (3D) scaffolds made of biopolymers or ECM molecules using bioprinting can be used to promote the wound healing process, especially for complex 3D tissue lesions like chronic wounds. Here, a 3D-printed mold has been designed to produce customizable collagen type-I sheets containing human umbilical vein endothelial cells (HUVECs) and adipose stromal cells (ASCs) for the first time. In these 3D collagen sheets, the cellular activity leads to a restructuring of the collagen matrix. The upregulation of the growth factors Serpin E1 and TIMP-1 could be demonstrated in the 3D scaffolds with ACSs and HUVECs in co-culture. Both growth factors play a key role in the wound healing process. The capillary-like tube formation of HUVECs treated with supernatant from the collagen sheets revealed the secretion of angiogenic growth factors. Altogether, this demonstrates that collagen type I combined with the co-cultivation of HUVECs and ACSs has the potential to accelerate the process of angiogenesis and, thereby, might promote wound healing.

## 1. Introduction

About 1% of the European population is affected by chronic wounds [1]. Chronic wounds are the result of an inadequate tissue repair process, leading to reduced quality of life, a high risk of infection, and increased treatment costs. Nowadays, it is well understood that wound healing is much more complex than just the four phases of hemostasis, inflammation, proliferation, and maturation [2]. It is a complex and well-orchestrated interplay of reactions and interactions between different cell types and mediators, e.g., growth factors, growth factor inhibitors, enzymes, as well as extracellular matrix (ECM) components [3].

Angiogenesis, the formation of new blood vessels, is a crucial step in the wound healing process, involving the chemotactic response of endothelial cells to macrophage-derived factors produced in the wound space [4,5,6]. During wound healing, the sprouting of angiogenic capillaries into the fibrin and fibronectin-rich wound clot occurs and, within a few days, organizes into a microvascular network [7]. It has been shown that there is a dynamic interaction between endothelial cells, angiogenic cytokines, such as FGF, VEGF, TGF-β, angiopoietin, and enzymes like mast cell tryptase and the ECM environment [8]. However, while TGF-β promotes angiogenesis in vivo, it inhibits cell growth and the proliferation of endothelial cell monolayers in vitro [9]. It has been shown that FGF-β plays an important role in angiogenesis during the first three days of wound repair, whereas VEGF has a crucial influence on angiogenesis from day four to seven during granulation tissue formation [10]. In addition to endothelial cells and angiogenic factors, the specific 3D ECM in the wound plays a key role in the wound healing process [11]. During the repair process, the ECM undergoes rapid changes and reformation as the fibrin clot is replaced by fibronectin and hyaluronan, followed by the substitution with collagen type I and III [11]. These changes are highly organized and tightly regulated both spatially and temporally [7].

For complex 3D tissue lesions such as chronic wounds, 3D scaffolds colonized with cells that stimulate cell adhesion, proliferation, and differentiation, guide migration, and eventually promote vascularization constitute a promising therapeutic approach [12,13,14,15]. In the early 1970s, it was suggested that with the support of new biocompatible materials, cells could be held in place in a synthetic scaffold structure, promote the synthesis of new ECM, and thereby accelerate the wound healing process [16].

3D scaffolds can be produced, for example, by traditional or additive manufacturing processes (e.g., selective laser sintering or 3D printing) [17]. After the preparation of the 3D scaffolds, the matrices are loaded with cells [18]. Conversely, bioinks containing cells embedded in a biocompatible hydrogel can be directly 3D-printed [19,20]. Because of the simultaneous processing of cells and biomaterials (e.g., ECM molecules), such constructs can be directly converted into tissue-like structures, and the maturation time can be minimized. However, many ECM molecules, such as e.g., collagen I, do not readily allow direct bioprinting due to their slow sol–gel transition and poor mechanical stability [21,22]. As an alternative, a temporal support structure that serves as a mold can be 3D-printed, and afterward, a biocompatible material containing cells and ECM molecules can be cast into the 3D-printed mold. Due to the 3D-printing process of the mold, the tissue construct can be designed to fit the wound of the patient after the mold is removed.

Human umbilical vein endothelial cells (HUVECs) undergo capillary-like tube formation when plated on a substrate [23,24]. Rocha and colleagues demonstrated that the assembly of vascular-like structures by HUVECs was improved in the presence of adipose stromal cells (ASCs), which shows the ability of these cells to reorganize the vascular milieu. The analysis of neuroregulatory factors showed that the co-cultivation of these cells upregulated the secretion of several neurotrophic factors relevant to trauma-related injuries [25].

The aim of this study was to generate a 3D collagen type-I scaffold with the potential to stimulate angiogenesis and wound healing. Therefore, for the first time, a 3D collagen scaffold was designed, which contained HUVECs and ASCs in co-culture. Through cell activity, the collagen matrix was largely reorganized from random fiber arrangement after six days of incubation to an aligned structure on day 19. To determine the angiogenic potential of the 3D scaffold, the obtained supernatant was evaluated using a 2D tube formation assay of HUVECs on a matrigel. Understanding the interaction between HUVECs, ASCs, and the collagen matrix during wound angiogenesis, particularly how the angiogenic growth factors affect the growth of the endothelial cells, is the basis for constructing customizable cell-laden collagen scaffolds. This could lead to future applications in the treatment of chronic wounds.

## 2. Materials and Methods

### 2.1. Cultivation of Human Umbilical Vein Endothelial Cells (HUVECs)

Human umbilical vein endothelial cells (HUVECs) (#C-12203, PromoCell GmbH, Heidelberg, Germany) were cultivated in Endothelial Cell Growth Medium 2 (PromoCell GmbH, Heidelberg, Germany) supplemented with 1% (*v*/*v*) antibiotic/antimycotic solution (Capricorn Scientific GmbH, Ebsdorfergrund, Germany) in a controlled atmosphere of 95% humidity, 5% CO_2_ at 37 °C. Cell viability and a number of cells were analyzed with the Casy TT Counter (Omni Life Science GmbH & Co. KG, Bremen, Germany).

### 2.2. Cultivation of Adipose Stromal Cells (ASCs)

Human adipose stroma cells (ASCs) (#PT-5006, Lonza Group Ltd., Basel, Switzerland) were cultivated in StemMACS™ MSC Expansion Media (Miltenyi Biotec, Bergisch Gladbach, Germany) supplemented with 1% (*v*/*v*) antibiotic/antimycotic solution (Capricorn Scientific GmbH, Ebsdorfergrund, Germany) in a controlled atmosphere of 95% humidity, 5% CO_2_ at 37 °C. Cell viability and a number of cells were analyzed with the CASY TT Counter (Omni Life Science GmbH & Co. KG, Bremen, Germany).

### 2.3. Analysis of Proliferation of 2D Co-Culture of ASCs and HUVECs

For cell proliferation analysis, 10,000 cells/cm^2^, either a co-culture of ASCs and HUVECs ratio 1:1 or only HUVECs were seeded on treated 24-well plates (TCP, Nunc, Thermo Fisher Scientific, Waltham, MA, USA) and cultivated for 19 days in a controlled atmosphere of 95% humidity, 5% CO_2_ at 37 °C. Every 2–3 days, the cells were washed twice with phosphate-buffered saline (PBS, Sigma Aldrich, Burghausen, Germany) and incubated with 10% (*v*/*v*) resazurin sodium salt (Sigma-Aldrich, St. Louis, MO, USA) in Endothelial Cell Growth Medium 2 (PromoCell GmbH, Heidelberg, Germany) supplemented with 1% (*v*/*v*) antibiotic/antimycotic solution (Capricorn Scientific GmbH, Ebsdorfergrund, Germany) for 2 h. Cell proliferation was quantified by measuring the fluorescence intensity of resorufin (λex 530 nm; λem 590 nm) using a plate reader (Mithras LB940 Berthold). The relative fluorescence is given in relative fluorescence units (rfu).

### 2.4. 3D Cell Culture in Collagen Sheets

For 3D cell culture experiments in engineered sheets, collagen type I was prepared according to the manufacturer’s protocol (L 7213, Biochrom AG, Berlin, Germany). Each collagen sheet consisted of a 115 µL collagen type-I solution laden with 20,000 cells, either a co-culture of ASCs and HUVECs every 10,000 cells or 20,000 HUVECs cells.

### 2.5. Preparation of 3D Collagen Sheets Using CAD and 3D-Printing

**Figure 1:**
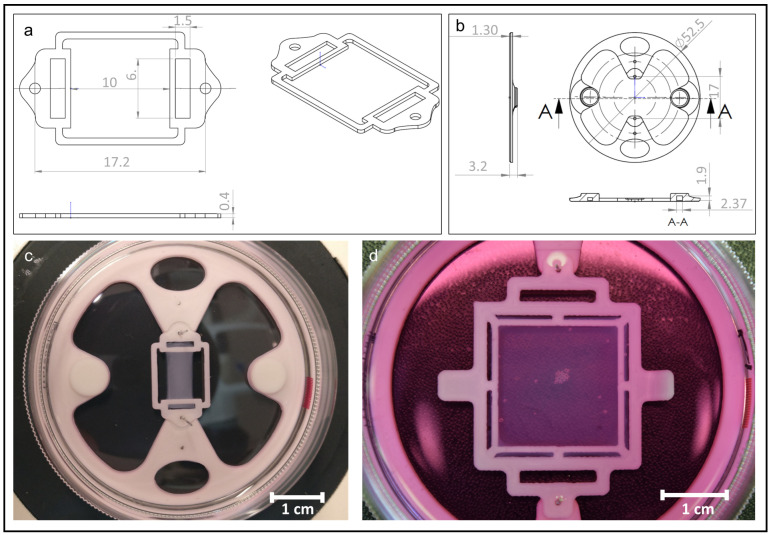
Scaffold design of collagen sheets. (**a**) Bracket design which was printed out of PLA (dimensions in mm); (**b**) baseplate made out of PLA for fixing the bracket’s position during cultivation and imaging (dimensions in mm); (**c**) cast collagen sheet attached to the bracket after the removal of the casting mold; (**d**) example for a customized scale-up of casted collagen sheet attached to PLA bracket and baseplate in Dulbecco’s Modified Eagle’s Medium.

The bracket and the base plate for collagen sheet fixation and the casting mold to cast the cell-laden collagen hydrogel in, were designed in SolidWorks (Dassault Systemes Solidworks Corp., Waltham, MA, USA). The dimensions of the bracket and the base plate, and the customizable casted collagen sheets are shown in Figure 1.

The support structure was fabricated using a fused deposition modeling Ultimaker 3 3D printer (Geldermalsen, The Netherlands) and a standard PLA thermoplast filament. (0.25 mm nozzle diameter, 0.1 mm layer thickness, 100% infill density, and 9 mm/s print speed). The baseplate was placed in a 35 mm standard lab Petri dish with magnets for adjustment. Afterward, the first layers of Pluronic F127 (0.33 g/mL) (Sigma Aldrich, Burghausen, Germany) were printed on a customized multi-purpose bioprinting platform directly into the Petri dish in the middle of the baseplate. The bracket was inserted in the middle of the baseplate, and the second layers of the pluronic sacrificial ink were printed on the bracket. The Pluronic casting mold was stored at 4 °C overnight. Collagen type I and the incorporated cells (ASCs and HUVECs) were cast into the mold and incubated for 2 h at 37 °C. The incubation times of the collagen sheets below 2 h resulted in very fragile sheets since collagen fibrillogenesis was not finished. Endothelial Cell Growth Medium 2 supplemented with 1% (*v*/*v*) antibiotic/antimycotic solution (PromoCell GmbH, Heidelberg, Germany) was used to dissolve pluronic at 37 °C overnight. Afterward, the media and pluronic were removed gently, and 8 mL of fresh cell culture media was added. To avoid potential damage to the engineered tissue, the cell culture media was only changed after the color change of the media and in combination with the cell culture supernatant sampling. For a period of 19 days, the supernatant was collected for further analysis and stored at −80 °C every 5–6 days. Afterward, the media was replaced with 8 mL of fresh media per Petri dish. Based on the size and shape of the baseplate, bracket, and casting mold customized collagen sheets can be produced.

### 2.6. Scanning Electron Microscopy (SEM)

SEM was performed using a JSM 6390 (JEOL, Tokyo, Japan) with a high-tension voltage of 10 kV. The samples were washed once with PBS and then fixed in 3% (*v*/*v*) glutaraldehyde at 4 °C for 2 days to analyze the collagen sheets. Afterward, the samples were washed again with PBS and dehydrated with ethanol 50%, 70%, 80%, 99% (*v*/*v*) for 30 min each step. The ethanol 99% (*v*/*v*) evaporated overnight, and the samples were sputtered with gold (BAL-TEC SCD 005) by vacuum evaporation for 40 s at 40 mA, 8 nm.

### 2.7. Sample Preparation for Atomic Force Microscopy (AFM)

To analyze the fibrous structure of the collagen sheets and the influence of the cell lines on the restructuring, the 3D collagen sheets were washed twice with PBS, then fixed 4 h at room temperature with 1.85% paraformaldehyde containing 10 µL/mL NaOH and 40 mg/mL sucrose. Afterward, the samples were washed three times with PBS and then incubated at 4 °C in 30% (*w*/*w*) sucrose. After 20 h, the samples were frozen in Tissue-Tek O.C.T. Compound (Sakura Finetek Germany GmbH, Staufen im Breisgau, Germany) using liquid nitrogen. The embedded collagen sheets were then mounted on a sample holder in such a way that the sheets could be sectioned parallel to the x–y plane defined by the sheets. As described previously [26], prior to every cut, adhesive tape was stuck to the sample, and 50 μm thick sections were sliced using a cryostat (Leica CM 1950, Leica Mikrosysteme Vertrieb GmbH, Wentzler, Germany). The sections on the adhesive tape were placed on frozen glass slides, on which double-sided adhesive tape had been placed beforehand, with the sheet section facing upwards. Sections were stored up to one day at −20 °C until investigation with the AFM.

### 2.8. AFM Measurements

The sections of the collagen sheets with the incorporated cells were thawed at room temperature directly before AFM measurements, which were carried out with a NanoWizard I (JPK Instruments, Berlin, Germany), as described previously [26,27,28,29]. In brief: Using an inverse optical microscope (Axiovert 200, Carl Zeiss MicroImaging GmbH, Munich, Germany), the silicon nitride cantilever (MLCT, Bruker, Bremen, Germany) with a nominal spring constant of 0.03 N/m and a four-sided pyramidal tip with a nominal radius of 20 nm was positioned at the area of interest. Images of different dimensions were recorded using contact mode imaging in the air near the initial mounting position, as well as at the center of the collagen sheets. Afterward, a droplet of standard phosphate-buffered saline (PBS, Dulbecco L1825, Biochrom GmbH, Berlin, Germany) was added to the section, and the collagen sheet was allowed to rehydrate for about 10 min. Indentation-type AFM (IT-AFM) measurements were then carried out in the same areas where the images were recorded before, each within a 30 µm × 30 μm grid containing at least 32 × 32 force–indentation curves. Two different sections were analyzed for every sheet (between 50 µm and 200 µm apart). For IT-AFM measurements, the same cantilevers were used as for AFM imaging. The optical lever sensitivity and actual spring constant of each cantilever were determined in triplicate using the thermal noise method in PBS and on a clean glass surface after each IT-AFM experiment [30].

### 2.9. AFM Data Analyses

AFM images were analyzed using JPK data analysis software (JPK Instruments, Berlin, Germany). The stiffness (Young’s modulus) of the collagen sheets was determined at each indentation position with a lab-made MATLAB application (Version R2018a, MathWorks, Inc., Natick, MA, USA) using the individually determined optical lever sensitivity and cantilever spring constant. The application automatically corrected for offset and tilt of the baseline and determined the contact point of each IT-AFM force curve [31]. Subsequently, a modified Hertz model for a four-sided pyramidal indenter, with a Poisson’s ratio of 0.5 and a tip half-opening angle to the edge of 17.5°, was fitted to the indentation part of the curves [29,32]. The whole indentation curve, including the contact point but omitting the first 500 nm, was fitted to extract the Young’s modulus at each position on the sheets. Force curves and fits were manually inspected and discarded if the quality of the curve did not allow for a reliable fit. Stiffness (Young’s modulus) distributions were plotted, and Gaussian distributions were fitted to them using IGOR Pro 8.03 (WaveMetrics, Inc., Portland, OR, USA).

### 2.10. Angiogenesis Array

For a screening of angiogenesis-related proteins, a Proteome Profiler Human Angiogenesis Array Kit (ARY007, R&D Systems Inc., Minneapolis, MN, USA) was used according to the datasheet, and pictures of the plot were taken with ChemiDoc Imaging System (Bio-Rad Laboratories Inc., Hercules, CA, USA). A volume analysis of the plot was performed with Image Lab 6.0 (Bio-Rad Laboratories Inc., Hercules, CA, USA). As a control, Endothelial Cell Growth Medium 2 (PromoCell GmbH, Heidelberg, Germany) was used. The data was analyzed using ANOVA one-way analysis of variance.

### 2.11. Tube Formation Assay

To evaluate the angiogenic potential of the samples, a tube formation assay was performed. A matrigel (reduced in growth factors; 354230, BD Biosciences; Olen, Belgium) was thawed on ice overnight, and pipette tips and angiogenesis slides (81506, ibidi GmbH, Gräfelfing, Germany) were precooled at 4 °C. An amount of 10 µL of matrigel was pipetted in every well of angiogenesis µ-slides and incubated for 1 h at 37 °C. HUVECs were cultured and counted as already described. Cells were resuspended in basal endothelial cell growth medium 2 without growth factors (PromoCell GmbH, Heidelberg, Germany), and 10,000 cells/well were added to the matrigel. Then, 40 µL of the supernatant, obtained from cell culture experiments in 3D collagen sheets, were added to each well. Endothelial Cell Growth Medium 2 (PromoCell GmbH, Heidelberg, Germany) served as positive control, and AIM V™ serum-free medium (12055091, Thermo Fisher Scientific, Waltham, MA, USA) served as a negative control. It was mixed gently and incubated in a controlled atmosphere of 95% humidity, 5% CO_2_ at 37 °C for 4 h. Afterward, two light microscope images were taken per well with Zeiss Axiovert 25 (Zeiss AG, Oberkochen, Germany) and angiogenic potential was analyzed with the software Wimasis (Onimagin Technologies SCA, Cardoba, Spain).

## 3. Results

### 3.1. Proliferation of 2D Co-Culture of ASCs and HUVECs

**Figure 2:**
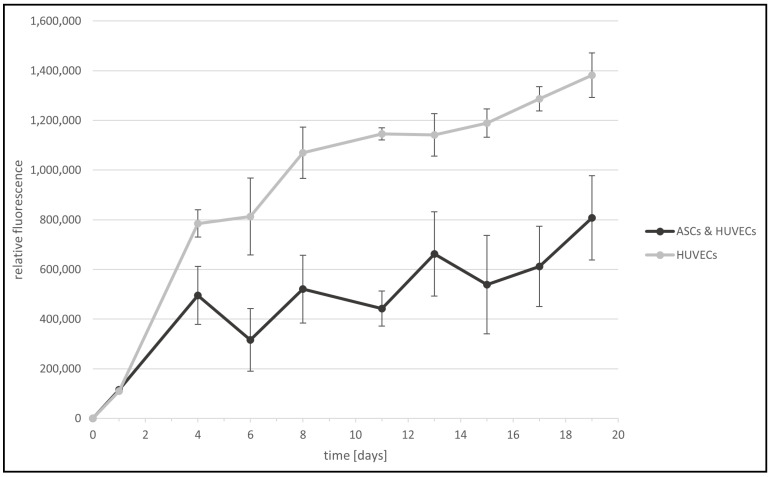
Cell proliferation of 2D cell cultures (HUVECs alone and HUVECs and ASCs in co-cultivation) was monitored by resazurin sodium salt assay. The relative fluorescence (rfu) of the cell cultures was analyzed over 19 days. Timepoint 0 represents the day of cell seeding. At this time point, no resazurin assay was performed and, therefore, the rfu is 0.

To analyze the viability of the cell lines, especially in co-cultivation, 10,000 cells/cm^2^ of human umbilical vein endothelial cells (HUVECs) alone as well as adipose stromal cells (ASCs) and HUVECs (ratio 1:1) in co-culture were cultivated over 19 days in a 24-well plate. Every 2–3 days, the cells were washed and then incubated with resazurin sodium salt for 2 h. The cell proliferation of both cell lines was analyzed using a resazurin sodium salt assay. Resazurin is a blue fluorogenic dye used as a redox indicator in cell viability and proliferation assays. The blue dye is irreversibly reduced by enzymes in viable cells to generate the red-fluorescent product, resorufin, which exhibits an emission maximum at ~595 nm and can be detected by fluorescence spectroscopy. The cell proliferation was quantified by measuring the fluorescence intensity of resorufin (Figure 2) [33].

Over 19 days, the relative fluorescence of ASCs and HUVECs in co-culture and HUVECs alone increased, and therefore, cell viability over time could be confirmed. HUVECs alone showed a relative fluorescence of 1,400,000 rfu, whereas HUVECs in co-cultivation with ASCs indicated a relative fluorescence of 800,000 rfu at 19 days. The increased relative fluorescence of HUVECs alone can be explained due to the small cell diameter of HUVECs in comparison to ASCs. Therefore, the total cell number and the resulting derived proliferation rate are higher. Nevertheless, the cultivation of HUVECs in the presence of ASCs showed proliferation and an increased relative fluorescence over time. The light microscopic analysis demonstrated that both cell lines had their typical cell morphology over time.

### 3.2. Restructuring of Collagen Sheets with HUVECs and ASCs Using Scanning Electron Microscopy (SEM)

**Figure 3:**
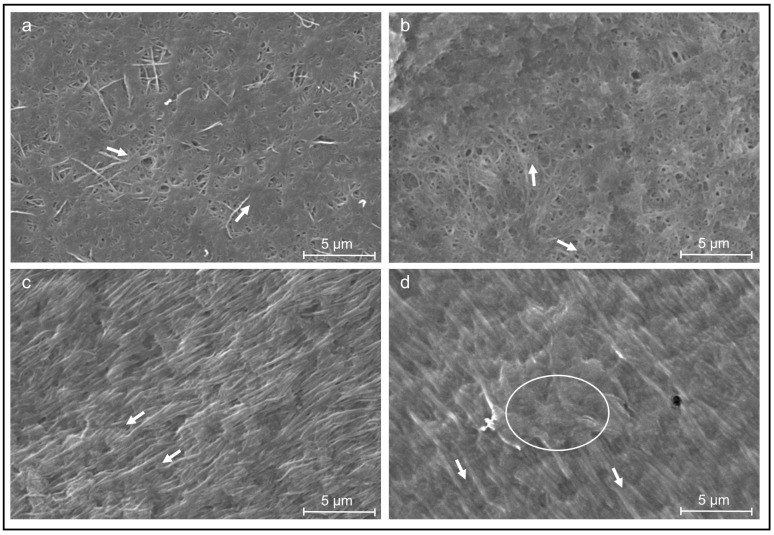
SEM images of collagen sheets seeded with different cell lines. (**a**) HUVECs and ASCs after six days of cultivation; (**b**) HUVECs after six days of cultivation; (**c**) HUVECs and ASCs after 19 days of cultivation; (**d**) HUVECs after 19 days of cultivation. Arrows: collagen fiber alignment. Circle: non-oriented collagen fibers.

To analyze the effect of both cell types on collagen fiber arrangement in 3D scaffolds, collagen sheets either laden with HUVECs or with ASCs and HUVECs were fabricated and cultivated for 19 days. The surfaces of these collagen sheets were examined by SEM after 6 and 19 days of incubation (Figure 3).

During the incubation period of 19 days, all of the cell types restructured the collagen matrix of the sheets. After six days of incubation, most collagen fibers are still randomly oriented, and only a few fibers are aligned and oriented parallel to each other in both cell cultures (Figure 3a,b). After 19 days of incubation, the collagen structures with HUVECs alone as well as in co-culture have changed and showed large areas of uniform fiber orientation, as shown in Figure 3c,d (white arrows indicate fiber orientation), coexisting with smaller areas, where the collagen fibers were not yet aligned (Figure 3d, white circle). Both cell cultures were able to restructure and superficially align the collagen fibers parallel to each other between day 6 and day 19. In addition, in the overview images (data not shown), all collagen sheets exhibited a shrinkage perpendicular to the axis defined by the anchoring points (as an example shown in Figure 1c). The shrinkage of the sheets is more distinct with the HUVECs in combination with the ASCs.

### 3.3. Atomic Force Microscopy (AFM) of Collagen Sheets

**Figure 4:**
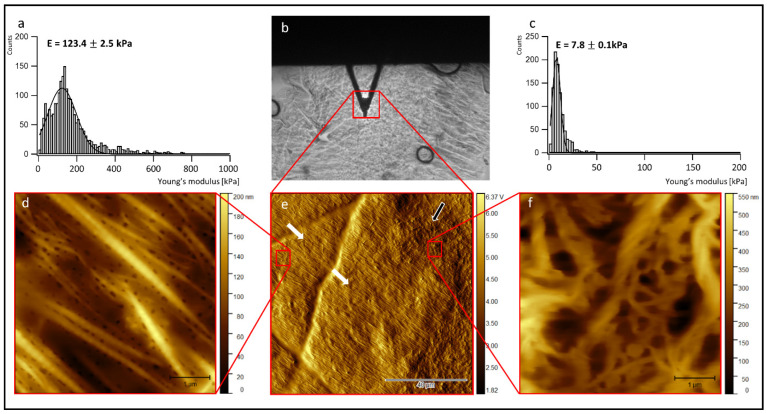
AFM measurement of cryo-sections from 3D collagen sheets laden with a co-culture of ASCs and HUVECs after 19 days of incubation. (**a**) Young’s modulus distribution in a region of aligned collagen fibers; (**b**) bright-field optical microscopy image, showing the collagen sheet (grey background), the AFM cantilever (dark triangle), and the region where the 100 µm × 100 µm overview image displayed below (**e**) was recorded; (**c**) Young’s modulus distribution in a region of randomly oriented collagen fibers; (**d**) contact mode AFM image (height image) in the region of aligned collagen fibers; (**e**) 100 µm × 100 µm AFM overview image (cantilever deflection image); (**f**) AFM image in the region of randomly oriented collagen fibers (height image). White arrows: aligned collagen fibers, black arrow: random fiber orientation.

To further analyze the structure inside the cell-laden collagen sheets and correlate it to its biomechanics, cryo-sections of the collagen sheets were prepared after 19 days of incubation, and AFM measurements were carried out on the thawed cryo-sections. First, contact mode AFM imaging in the air was performed to investigate the sheet structure, including the collagen fiber orientation and the ultra-structure of the collagen fibers. To correlate the observed structural features to the biomechanical properties of the cell-laden collagen sheets, indentation type AFM measurements (IT-AFM) were carried out on selected areas of the rehydrated cryo-sections in a second step (see Section 2 for details of sample preparation for AFM-imaging and IT-AFM). Figure 4 summarizes representative AFM results of a cryo-sectioned collagen sheet containing a co-culture of ASCs and HUVECs.

In the center of the top row (Figure 4b), a bright-field optical microscopy image shows the collagen sheet (grey background), the AFM cantilever (dark triangle), and the region, where a first 100 µm × 100 µm AFM overview image was recorded (red square). The image below (Figure 4e) shows the corresponding 100 µm × 100 µm AFM overview image. This overview image exhibits large areas of highly ordered parallel collagen fibers (left and central part of the image, white arrows), as well as smaller areas showing random collagen orientation (right part of the image, black arrow). A 5 µm × 5 µm close-up (Figure 4d) of the upper left part of Figure 4e (red square on the right side) indeed reveals homogeneously oriented, highly parallel collagen fibers. In the areas of this close-up, even the 67 nm D-band structure of the collagen fibers becomes visible if the contrast of the color table is enhanced (see Figure S1a in the Supplementary Materials). A 5 µm × 5 µm close-up (Figure 4f) of the upper right part of Figure 4e (red square on the left side) shows randomly oriented collagen fibers with a broad range of fiber diameters and fibrillar substructures. Here, no clear D-band structure is detectable.

Interestingly, the Young’s modulus distribution recorded in the region with parallel collagen orientation Figure 4a) peaks at an almost 16× higher value (123.4 kPa) than the Young’s modulus distribution recorded in the area with random collagen orientation (7.8 kPa) (Figure 4c). Note that the absolute Young’s modulus values do not represent the values of the native collagen sheets, as the sheets were fixed with PFA before cryo-sectioning (see Section 2 for details). However, the Young’s modulus differences observed between the two areas reflect the underlying structural differences in the two different areas of ordered and randomly oriented collagen fibers.

**Figure 5:**
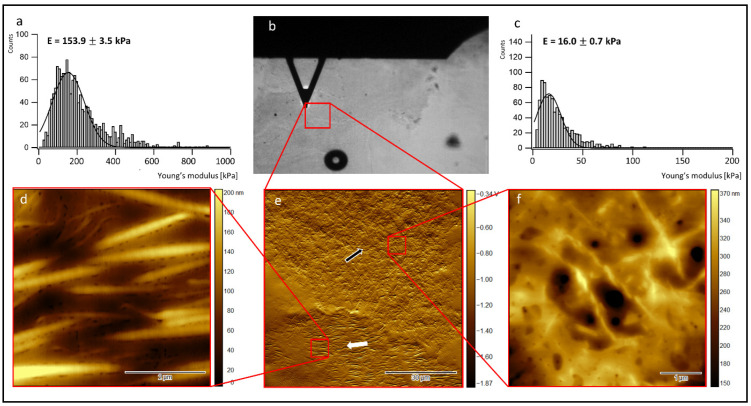
AFM measurement of cryo-sections from collagen sheets laden with HUVECs after 19 days of incubation. (**a**) Young’s modulus distribution in a region of aligned collagen fibers; (**b**) bright-field optical microscopy image, showing the collagen sheet (grey background), the AFM cantilever (dark triangle), and the region where the 100 µm × 100 µm overview image displayed below (**e**) was recorded; (**c**) Young’s modulus distribution in a region of randomly oriented collagen fibers; (**d**) contact mode AFM image (height image) in the region of aligned collagen fibers; (**e**) 100 µm × 100 µm AFM overview image (cantilever deflection image); (**f**) AFM image in the region of randomly oriented collagen fibers (height image). White arrow: aligned collagen fibers, black arrow: random fiber orientation.

Figure 5 summarizes the AFM results of a representative cryo-section obtained from a HUVEC-containing collagen sheet.

The center of the top row (Figure 5b) again shows a bright-field optical microscopy image of the collagen sheet (grey background), the position of the AFM cantilever (dark triangle), and the region where a first 100 µm × 100 µm AFM overview image was recorded (red square). The image below (Figure 5e) shows the 100 µm × 100 µm AFM overview image of the cell-laden sheet. This overview image again exhibits areas of highly ordered parallel collagen fibers (lower part of the image, white arrows), as well as areas showing random collagen orientation (central and upper part of the image, black arrow). The 5 µm × 5 µm close-up (Figure 5d) of the lower left part of Figure 5e (red square on the lower left side) again shows homogeneously oriented, highly parallel collagen fibers. Here, the 67 nm D-band structure is again visible if the contrast of the color table is enhanced (data not shown). The 5 µm × 5 µm close-up (Figure 5f) of the upper right part of Figure 5e (red square on the upper right side) shows randomly oriented collagen fibers with various fiber diameters and no detectable D-band structure. The Young’s modulus distribution in the region with parallel collagen orientation (Figure 5a) peaks at an almost 10× higher value (153.9 kPa) than the Young’s modulus distribution (Figure 5c) in the area with random collagen orientation (16.0 kPa), reflecting the structural differences in the chosen areas.

### 3.4. Angiogenic Potential of the 3D Collagen Sheets

An angiogenic array and a tube formation assay were performed to analyze the angiogenic potential of the cell-laden 3D collagen sheets.

#### 3.4.1. Angiogenic Array of the Cell Culture Supernatant Collected from Cell-Laden 3D Collagen Sheets

**Figure 6:**
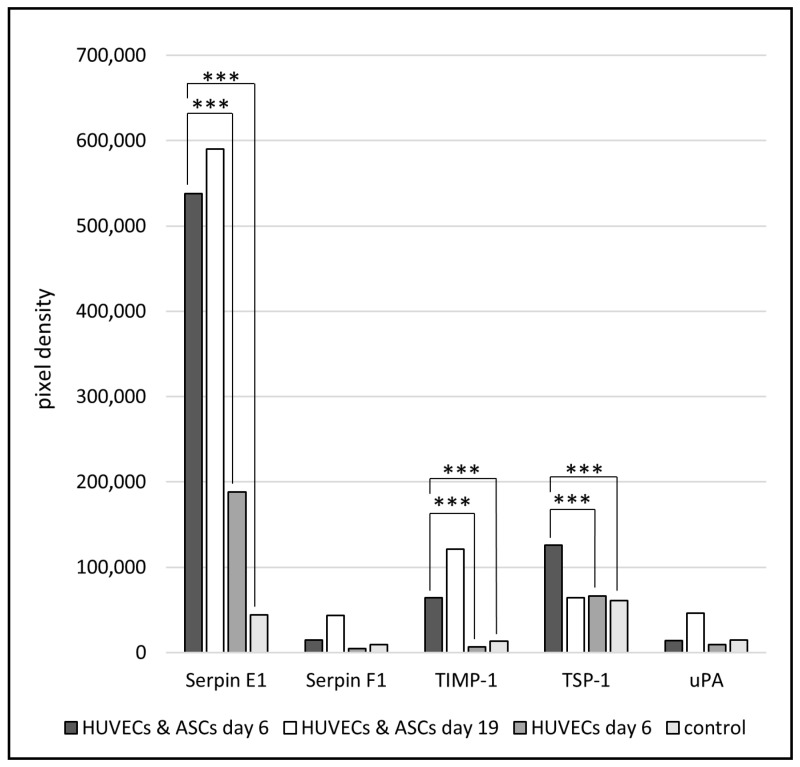
Expression of selected angiogenic factors in cell-laden collagen sheets. Cell culture supernatants were collected after 6 and 19 days of cultivation and analyzed using the Proteome Profiler Human Angiogenesis Array Kit for potential growth factors. As a control, Endothelial Cell Growth Medium 2 (containing angiogenic growth factors) was used to validate the assay. The * shows the significant difference between the values. Therefore, the data were analyzed using one-way Analysis of Variance (ANOVA). Probability values (***) *p* < 0.001 were considered the most significant.

For profiling angiogenesis-related factors, the supernatants of cell-laden collagen sheets were collected on day 6 and day 19 and analyzed using the proteome profiler human angiogenesis array kit. An overview of the results of this array is shown in Figure 6.

Serpin F1 and urokinase-type plasminogen activator (uPA) showed only a slight difference between HUVECs and the co-culture of ASCs and HUVECs after 6 days and a further slight increase at day 19. However, after 6 days, Serpin E1, TIMP 1, and TSP-1 are the most significantly upregulated in the co-culture of ASCs and HUVECs in comparison to HUVECs alone and the control. After 19 days of the co-culture, Serpin E1 and TIMP-1 showed an additional increase compared to day 6. As a control, Endothelial Cell Growth Medium 2 was used and always showed the lowest level of growth factors. However, TSP-1 is downregulated between days 6 and 19 in the collagen sheets containing ASCs and HUVECs.

#### 3.4.2. HUVECs Tube Formation Assay of the Cell Culture Supernatant Obtained from Co-Culture Experiments

**Figure 7:**
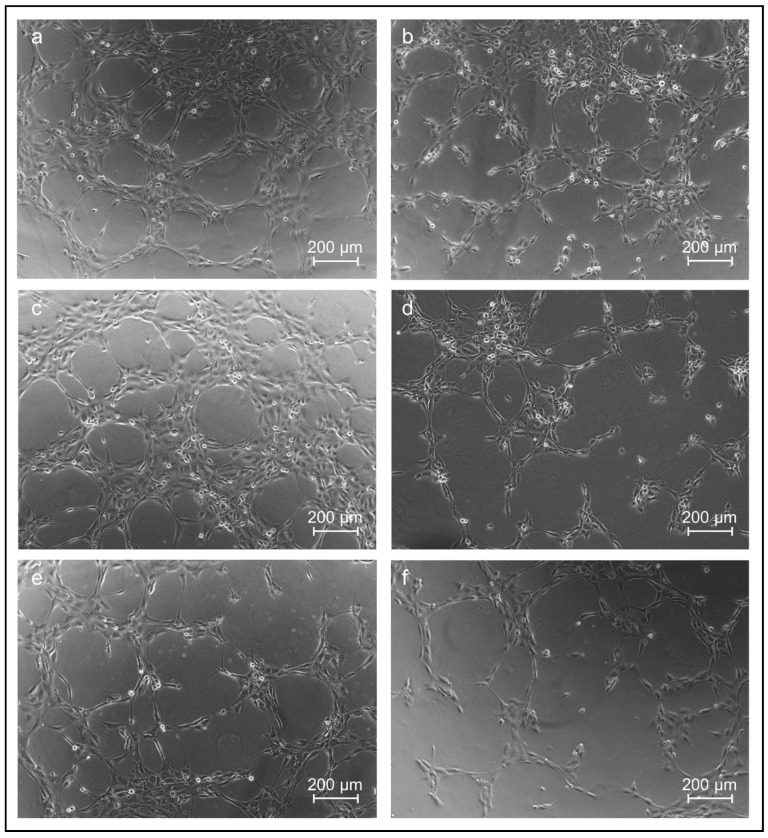
Bright-field microscopy images of the HUVECs tube formation assay after 4 h of incubation with supernatants obtained from the cultivation of different cell-laden 3D collagen sheets. (**a**) Treated with supernatant from 3D construct laden with HUVECs and ASCs after six days of cultivation; (**b**) Treated with supernatant from 3D HUVECs construct after six days of cultivation; (**c**) Treated with supernatant from 3D HUVECs and ASCs construct after 19 days of cultivation; (**d**) Treated with supernatant from 3D HUVECs construct after 19 days of cultivation; (**e**) positive control cultivated in Endothelial Cell Growth Medium 2 containing angiogenic growth factors; (**f**) negative control using AIM V cytokine and serum-free medium for incubation.

The 2D tube formation assay with HUVECs enables a statement about the angiogenic potential of the collected cell culture supernatant from the collagen sheets. Therefore, the cell culture supernatant obtained from the previous experiments with co-cultured HUVECs and ASCs, as well as HUVECs alone in 3D collagen sheets, was incubated with HUVECs (10,000 cells/well) seeded on matrigel (with reduced growth factors) on angiogenesis slides. Endothelial Cell Growth Medium 2 was used as positive control and AIM V serum-free medium as a negative control. The representative bright-field microscopy images are shown in Figure 7.

HUVECs treated in the tube formation assay with the supernatant of HUVECs and ASCs co-cultured in the collagen sheets over 6 and 19 days, respectively, showed a remarkable tube formation (Figure 7a,c). Here, the cells arranged themselves in such a way that more closed tubes and more branching points occurred in comparison to HUVECs treated with the supernatant of HUVECs alone (Figure 7a–d). The treatment with HUVEC and ASC supernatant (19 days) was comparable to the positive control, where the HUVECs were treated with Endothelial Cell Growth Medium 2 (Figure 7e), whereas the negative control with serum-free medium indicated a reduced tube formation (Figure 7f). The supernatant obtained from the cultivation of HUVECs alone in collagen sheets induced less tube formation compared to the co-cultivation (Figure 7a–d).

**Figure 8:**
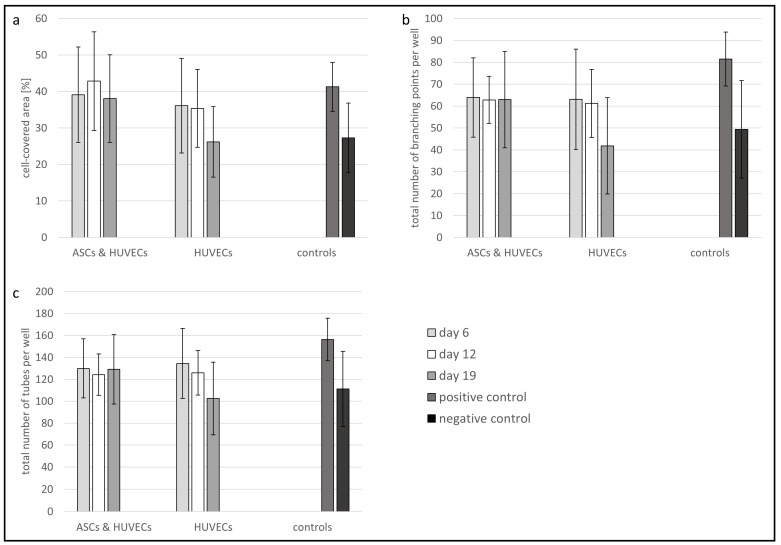
Quantitative analysis of the tube formation assay. (**a**) Cell-covered area per well; (**b**) total number of branching points per well; (**c**) total number of tubes per well. The cell culture supernatants were collected after different time points (days 6, 12, and 19) to investigate their angiogenic potential. Error bars indicate the standard deviation.

To quantify the tube formation assay, the area covered by tubes (Figure 8a), the total number of branching points of the tubes (Figure 8b), and the total number of the formed tubes per well (Figure 8c) were determined using the image analysis software Wimasis.

Figure 8 shows that the supernatants, which were obtained from collagen sheets laden with HUVECs and ASCs as well as supernatants from sheets laden with HUVECs alone, cause a comparable tube formation effect in the 2D tube formation assay: HUVECs treated with supernatant from co-culture sheets containing ASCs and HUVECs, show a slightly higher covered area (Figure 8a) at day 6 and day 12 than HUVECs treated with supernatant obtained from only-HUVEC-laden sheets (covered area of approximately 35%). The number of branching points (Figure 8b) and the total number of tubes (Figure 8c) induced in the tube formation assay are nearly identical for both supernatants on day 6 and day 12. On day 19, the values of all the indicators for tube formation fall to the level of the negative control for supernatants from HUVEC-laden sheets, while the tube formation potential of supernatants obtained from co-cultures remains the same (Figure 8). These investigations indicate a comparable angiogenic potential of supernatants obtained from 3D collagen sheets laden with co-cultures of ACS and HUVECs and supernatants from HUVEC-laden sheets until day 12. On day 19, this potential is reduced in supernatants obtained from the HUVEC-laden sheet, whereas it remains unchanged in supernatant from co-cultures. Note that the changes in the values shown in Figure 8 are not statistically significant.

## 4. Discussion

The wound healing process is a complex interplay between different cell lines and mediators such as growth factors and cytokines as well as ECM components [8]. For the regeneration of complex 3D tissues, such as chronic wounds, 3D scaffolds colonized with cells that facilitate the infiltration of cells as well as enable cell adhesion, migration, and proliferation show significant potential [12,13]. The synthesis of new ECM components is promoted, and finally, the wound healing process is accelerated.

In this study, customizable collagen type I sheets containing a co-culture of HUVECs and ASCs were fabricated and evaluated for their potential to stimulate angiogenesis. For the production of the collagen sheets, 3D-printed Pluronic molds, as well as 3D-printed base layers and brackets made of PLA, have been used as templates. Due to the 3D printing of the molds, these constructs can be easily adapted to the wound geometry of the patient.

The 3D ECM plays a key role in the wound healing process. The major components of the ECM are fiber-forming proteins, such as collagens, elastin, fibronectin, laminin, glycoproteins, proteoglycan, and glycosaminoglycans [34]. Among these ingredients, collagen is the most abundant, forming large fibrillar structures and providing mechanical stability [35]. Together with other ECM proteins and proteoglycans, it forms a complex 3D matrix [36]. This matrix supports cells and newly forming blood vessels [37,38]. In the wound healing process, especially in chronic wounds, the role of collagen is important due to its chemotactic ability [39]. It attracts fibroblasts and keratinocytes to the wound, leading to the deposition of new collagen in the wound and angiogenesis and reepithelialization [40,41]. Collagen is biodegradable, biocompatible, and can be easily gained and modified [42]. Therefore, a 3D in vitro approach made of collagen was established to improve the angiogenic environment for blood vessel-forming cells. Furthermore, the 3D scaffold should provide an environment where cells can survive, migrate and proliferate.

Ramanathan et al. showed that NIH 3T3 fibroblasts and human keratinocytes (HaCaT) assisted in excellent cell adhesion and proliferation in a 3D collagen scaffold. The collagen was combined with a bioactive extract to reduce the infection at the wound site [43]. Recently, it has also been demonstrated that human fibroblasts show normal cell growth within wound dressings based on collagen and essential oil functionalized ZnO nanoparticles [44]. In our study, HUVECs and ASCs were incorporated into the 3D collagen sheets to demonstrate the interaction between both cell lines and the secretion of angiogenic growth factors. ASCs secrete various angiogenic cytokines and growth factors and may be used to promote vascularization and growth of tissues and accelerate the wound healing processes [45]. In 2020, Rocha et al. demonstrated a synergistic effect of ASCs and HUVECs in a gellan gum hydrogel with a high potential for treating spinal cord injuries. HUVECs had only the ability to form vascular-like structures in the presence of ASCs [25].

In the current study, it was demonstrated that HUVECs could also proliferate in the presence of ASCs over 19 days. In the process of wound healing, the ECM undergoes rapid changes, e.g., the fibrin clot is replaced by fibronectin and hyaluronan, followed by the substitution of collagen type I and III. Therefore, the collagen matrix is permanently reorganized [46]. The restructuring of the originally randomly oriented casted collagen into a highly structured collagen matrix with highly oriented collagen fibrils in large areas of the sheets could be demonstrated in our in vitro approach, using both SEM and AFM. While the SEM micrographs (Figure 3) focus on the collagen micro-structure at the outer surface of the cell-laden collagen sheets and highlight changes observed between days 6 and 19 of cultivation, the AFM micrographs of cryo-sections of the sheets (Figure 4 and Figure 5) display their internal structure. The AFM micrographs also provide access to the ultra-structure of the sheets, exhibiting the well-known 67 nm D-band structure along the collagen fibrils [47]. In addition, the indentation measurements carried out with the AFM (summarized in Figure 4a,c and Figure 5a,c) allow for the correlation of the differences observed in the collagen structure and orientation to the nanomechanical properties of the sheets. As expected, the Young’s modulus is significantly higher in the structured and oriented areas of the collagen sheets [26].

Using the angiogenesis array, the impact of ASCs on HUVECs through the upregulation and the secretion of growth factors, like Serpin E1, TIMP-1, and TSP-1, could be demonstrated. Serpins participate in almost all stages of wound repair, where they regulate coagulation, fibrinolysis as well as inflammation [48]. At the same time, the TSP-1 was downregulated over time in the co-culture supernatant. In addition, the TSP-1 concentration was determined using an Enzyme-linked immunosorbent assay (ELISA). The concentration was found to be below the detection limit of the used EILSA kit (Supplementary Materials). TSP-1 directly inhibits angiogenesis by effecting endothelial cell proliferation, migration, and survival [49]. The most critical step in the wound healing process is the supply of the newly formed tissue with oxygen and essential nutrients. Therefore, angiogenesis, i.e., the formation of new blood vessels in the wound, is mandatory. The angiogenic potential can be analyzed by a tube formation assay, where capillary-like structures are formed by endothelial cells in response to the presence of angiogenic growth factors [50]. In our study, the tube formation assay confirmed the angiogenic potential of the collagen sheets containing HUVECs and ASCs. Whereas the endothelial cells treated with supernatant from collagen sheets containing HUVECs alone for 19 days demonstrated a decreased cell-covered area and the total number of branching points as well as a reduced total number of tubes. Therefore, the released growth factors of ASCs showed a positive effect on the capillary-like tube formation of HUVECs.

Altogether, the designed, customizable 3D collagen sheets containing HUVECs and ASCs in co-cultivation are encouraging, and, in future investigations, these cell-laden collagen sheets might be used as a potential treatment for chronic wounds to improve the wound healing process.

## 5. Conclusions

Cell-laden collagen type I hydrogels could be cast into 3D sheets and cultivated over 19 days. Both embedded cell lines, adipose stromal cells (ASCs) and human umbilical vein endothelial cells (HUVECs), were able to survive and proliferate in the engineered collagen scaffolds. It could be demonstrated that both cell lines can lead to a reorganization and restructuring of the collagen fibers of the scaffold. There is an interaction between collagen, one of the main components of the extracellular matrix, ASCs, and HUVECs, and the growth factors can act as mediators for wound healing processes. Over 19 days, the ASCs released different growth factors (e.g., Serpin E1 and F1, TIMP-1 and uPA). These growth factors enhanced the tube formation of the HUVECs and thereby showed a high potential for angiogenesis, a critical step in the wound healing process. The customizable collagen sheets affect cell behavior and enhance angiogenesis. In addition, they can be adapted to the wound of the patient. This in vitro model has potential for angiogenesis but needs further optimization in vitro; a forthcoming in vivo evaluation will provide additional information.

## Data Availability

The data presented in this study are available on request from the corresponding author.

## Supplementary Materials

The following supporting information can be downloaded at: https://www.mdpi.com/article/10.3390/jfb13030107/s1, Figure S1: D-band structure.
