# Supplementary material for: Angiogenic Potential of Co-Cultured Human Umbilical Vein Endothelial Cells and Adipose Stromal Cells in Customizable 3D Engineered Collagen Sheets
Source: J Funct Biomater. 2022 Jul 29;13(3):107. doi: 10.3390/jfb13030107 (PMC9397038; doi:10.3390/jfb13030107)
Supplement: Supplementary file 1 [file jfb-13-00107-s001.zip › jfb-1804432-supplementary.pdf]

Supplemental Information

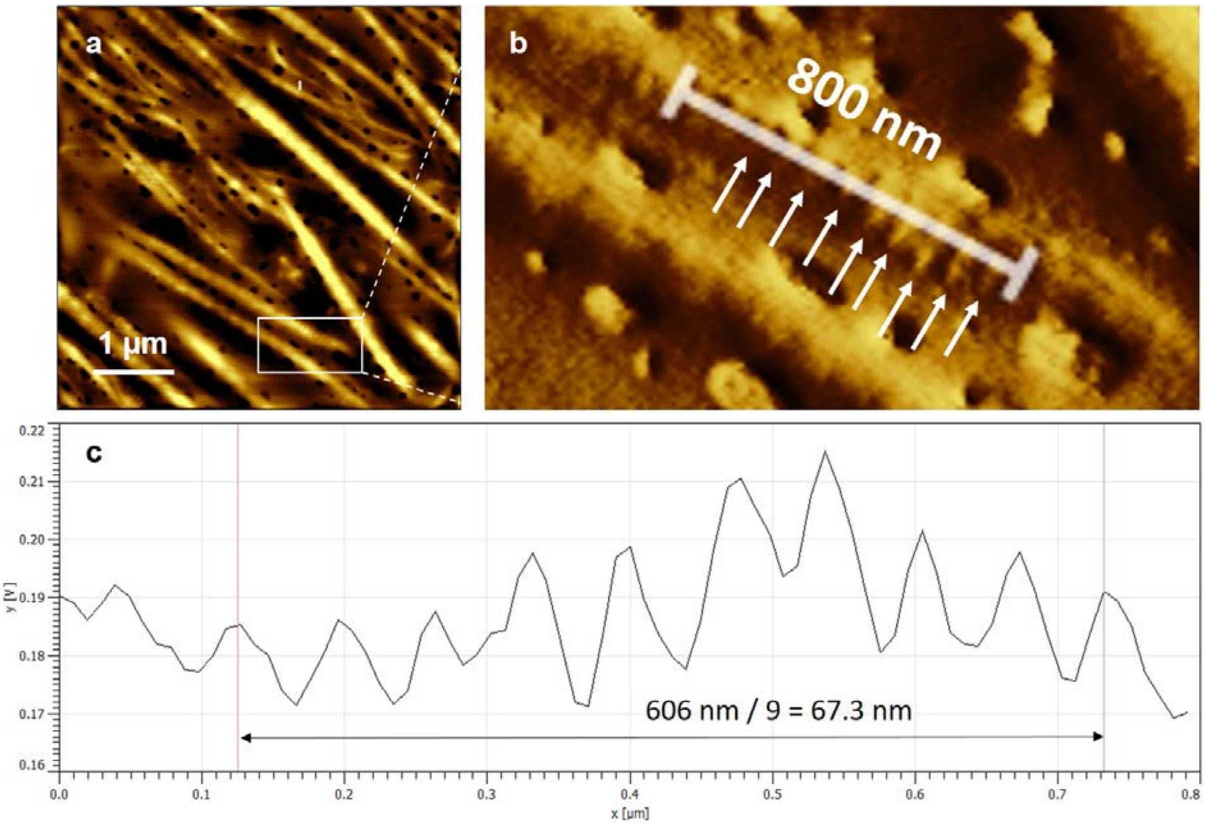

Figure S1. D-band structure. **(a)** The 5 µm x 5 µm close-up image is shown in Figure 5d with enhanced contrast of the color table; **(b)** vertical cantilever deflection image of the area indicated by the white rectangle in (a), showing the periodic 67 nm D-band structure of collagen (white arrows); **(c)** profile along the 800 nm long white line indicated in (b). Here the D-band structure with a periodicity of ≈ 67 nm is clearly recognizable.

Supplemental methods

Enzyme-linked immunosorbent assay (ELISA) of Human Thrombospondin-1 (TSP-1)

To quantify the concentration of TSP-1 over time, in addition to the angiogenic array, an enzyme-linked immunosorbent assay (ELISA) was performed. The previously taken supernatant samples were used to determine the concentration of TSP 1 in the cell culture media over time. Therefore, a Human Thrombospondin-1 DuoSet ELISA (DY3074, R&D Systems Inc., USA) was performed according to the datasheet and measured with a plate reader (Mithras LB940 Berthold). The used ELISA kit had a detection limit of 1.6 ng/mL. The measured concentration of TSP-1 was not detectable in the ELISA and was therefore below 1.6 ng/mL over 19 days in both groups (data not shown).
